# Antimicrobial Resistance Markers of Class 1 and Class 2 Integron-bearing *Escherichia coli* from Irrigation Water and Sediments

**DOI:** 10.3201/eid0907.020529

**Published:** 2003-07

**Authors:** Matthew T. Roe, Everardo Vega, Suresh D. Pillai

**Affiliations:** * Texas A&M University, College Station, Texas, USA

**Keywords:** Integrons, river, irrigation water, sediments, *E. coli*, phenotype, antimicrobial resistance, aminoglycoside, streptomycin, gene cassette, research

## Abstract

Municipal and agricultural pollution affects the Rio Grande, a river that separates the United States from Mexico. Three hundred and twenty-two *Escherichia coli* isolates were examined for multiple antibiotic resistance phenotypes and the prevalence of class 1 and class 2 integron sequences. Thirty-two (10%) of the isolates were resistant to multiple antibiotics. Four (13%) of these isolates contained class 1–specific integron sequences; one isolate contained class 2 integron–specific sequences. Sequencing showed that the class 1 integron–bearing strain contained two distinct gene cassettes, *sat-1* and *aadA*. Although three of the four class 1 integron–bearing strains harbored the *aadA* sequence, none of the strains was phenotypically resistant to streptomycin. These results suggest that integron-bearing *E. coli* strains can be present in contaminated irrigation canals and that these isolates may not express these resistance markers.

Integron gene sequences contribute to the spread of antimicrobial resistance alleles by lateral gene transfer of gene cassettes in a variety of enteric bacteria, including *Campylobacter* spp., *Escherichia coli,* and *Salmonella enterica* serotype Typhimurium (*1*–*4*). The gastrointestinal environment is suspected of serving as a reservoir for integron-bearing strains; when antimicrobial exposure occurs, gene transfer events—which spread cassettes between commensal organisms that are expelled into the environment (*2*)—would also occur.

The Rio Grande, the river separating the United States from Mexico along the Texas-Mexico region, serves as a source for irrigation water in Texas and Mexico. Previous studies in our laboratory and others have shown that the transboundary region is subject to extensive microbial and chemical contamination. This contamination has been associated with agricultural, municipal, and industrial wastes originating from both sides of the border (*5*,*6*). Leaking septic tanks and wastewater effluent discharges result in fecal contamination levels as high as 2,000 CFU/mL of fecal coliforms (*7*,*8*).

Because of the strategic importance of the Rio Grande for U.S. agriculture and the potential transmission of antimicrobial resistance determinants by means of food crops, we investigated the prevalence and characteristics of class 1 and class 2 integron–bearing *E*. *coli* strains. These strains were previously isolated from a study investigating fecal contaminants in irrigation water and associated sediments at specific locations along the river (*9*).

## Methods

Three hundred and twenty-two *E. coli* isolates were previously isolated from irrigation water and associated sediments at the El Paso, Presidio, and Weslaco regions of the river (*9*). After being confirmed as *E. coli* by MUG (4-methyl umbelliferyl-β-D-glucoronide)–based fluorescence, these isolates were screened for antimicrobial susceptibility by using the agar dilution method (*10*,*11*). The isolates were tested against ampicillin, tetracycline, ceftriaxone, cephalothin, gentamicin, kanamycin, streptomycin, chloramphenicol, ciprofloxacin, and trimethoprim/sulfamethoxazole. The antibiotics were tested at concentrations established by the National Antimicrobial Resistance System (*12*).

Isolates that were multidrug resistant (resistant to two or more antimicrobial agents) were grown overnight in 5 mL of Mueller-Hinton broth (Accumedia, Baltimore, MD) with the appropriate concentration of antimicrobial compound. A 1-mL aliquot of the culture was centrifuged at 10,000 rpm for 2 min. The cell pellet was resuspended in 500 μL of sterile water and boiled for 10 min. The resulting DNA suspension was used as template DNA in polymerase chain reaction (PCR) amplification for the class 1 and class 2 integrase gene and variable regions using the primer sequences shown in the Table (*13*–*15*).

The PCR reactions used 10 μL of template DNA, 5 µM of primers, 25 mM MgCl, 10 mM deoxynucleotide triphosphate, and 23 ng bovine serum albumin. Nuclease-free water (Ambion, Austin, TX) was added to achieve a volume of 50 μL. A “hot start” method was used, and 1.25 U of *Taq* DNA polymerase (Sigma, St. Louis, MO) was added after initial template denaturation. The PCR cycle was as follows: initial denaturation for 12 min at 94°C, hot start pause at 80°C followed by 35 cycles of denaturation at 94°C for 1 min, primer annealing at 60°C for 1 min, and extension at 72°C for 5 min at first cycle. An additional 5 s was progressively added to each cycle to reach a final of 7 min, 55 s. PCR products were analyzed on 1% agarose gel.

Amplification products were extracted from the gels with the QIAGEN QIAquick gel extraction kit (Valencia, CA). The amplified products were sequenced at a commercial facility (MWG Biotech Inc., High Point, NC) with the class 1 and class 2 integron variable region primers (*integ* and *hep*) (Table). Contiguous sequences were created from single sequence reads by using the CAP3 sequence assembly program (*16*). Contiguous sequences were analyzed by using the GenBank database of the National Center for Biotechnology Information and the BLASTX search engine (*17*). Putative gene relationships and sequence data were analyzed by using a multiple sequence alignment created by using Clustal W version 1.82 (*18*).

**Table:** Oligonucleotide primer sequences used for amplification of class 1 and class 2 integrase and variable regions




Primer	Primer sequence	Target	Reference

integ-1	5'-GGCATCCAAGCAAG-3'	5'-Class 1 integron variable region	Levesque et al. 1995 (*13*)
integ-2	5'-AAGCAGACTTGACCTGA-3'	3'-Class 1 integron variable region	Levesque et al. 1995 (*13*)
hep51	5'-GATGCCATCGCAAGTACGAG-3'	5'-Class 2 integron variable region	White et al. 2001 (*14*)
hep74	5'-CGG GATCCCGGACGGCATGCACGATTT GTA-3'	3'-Class 2 integron variable region	White et al. 2001 (*14*)
intI1.F	5'-GGGTCAAGGATCTGGATTTCG-3'	5'-*intI1* gene	Mazel et al. 2000 (*15*)
intI1.R	5'-ACATGGGTGTAAATCATCGTC-3'	3'-*intI1* gene	Mazel et.al. 2000 (*15*)
intI2.F	5'-CACGGATATGCGACAAAAAGGT-3'	5'-*intI2* gene	Mazel et al. 2000 (*15*)
intI2.R	5'-GTAGCAAACGAGTGACGAAATG-3'	5'-*intI2* gene	Mazel et al. 2000 (*15*)

## Results

**Figure 1:**
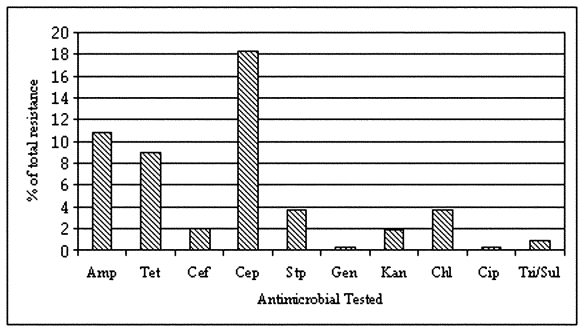
Frequency of antimicrobial resistance observed in 322 *Escherichia coli* isolates from irrigation water along the Texas-Mexico border.

Of the 322 *E. coli* isolates from sediment and irrigation water samples analyzed for antimicrobial resistance, 104 (32%) isolates showed resistance to at least one of the antimicrobial compounds (Figure 1). Approximately 10% (32/322) of all the isolates showed a multidrug resistance phenotype. Eighteen percent of the isolates were resistant to cephalothin; however, only 5 (2%) of 322 were resistant to ceftriaxone, which also belongs to the cephalosporin family. Resistance to ampicillin was prevalent in approximately 35 (11%) of the isolates. Resistance to tetracycline (9%), kanamycin (2%), gentamicin (0.3%), and streptomycin (4%) was also observed. Resistance to the fluoroquinolone ciprofloxacin was seen in one isolate. Three (<1%) of the 322 isolates were resistant to sulfonamide sulfamethoxazole. On the basis of analysis of variance, antimicrobial resistance and the sampling location were correlated. Isolates from the El Paso sampling region had significantly higher (p<0.05) antimicrobial resistance as compared with the Presidio and Weslaco sampling regions (data not shown).

**Figure 2:**
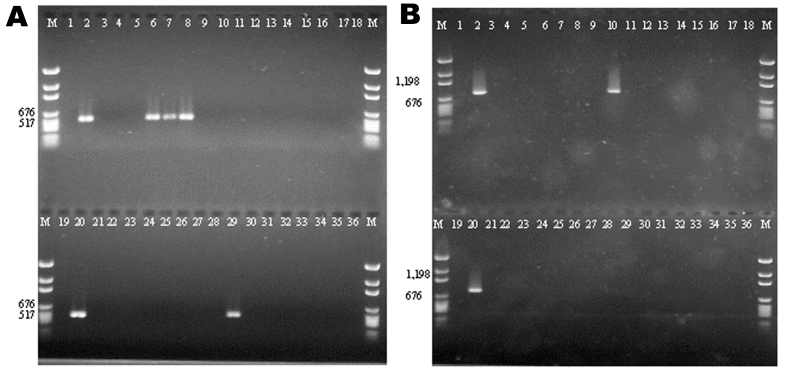
Agarose gel electrophoresis of integrase gene polymerase chain reaction (PCR) amplification products. A: PCR products of class 1 integrase gene *intI1*. Lane M; molecular marker; lanes 1 and 19: no template (negative) control; lanes 2 and 20: positive control (In2); lanes 3–36: multiple drug–resistant isolates. B: PCR products of class 2 integrase gene *intI2*. Lane M: molecular marker; lanes 1 and 19: no template control (negative) control; lanes 2 and 20: positive control (Tn7); lanes 3–36: multidrug-resistant isolates.

**Figure 3:**
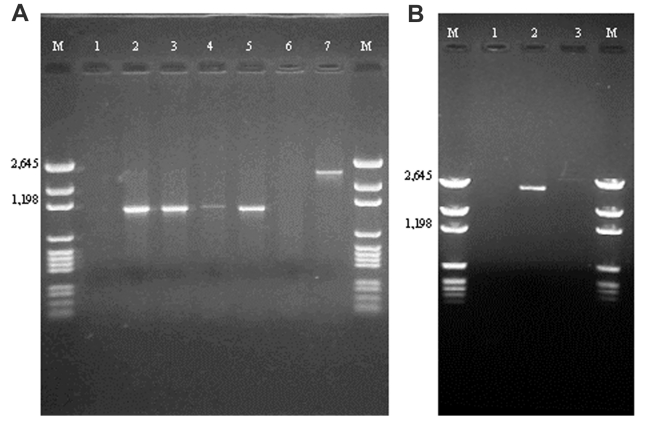
A: Polymerase chain reaction (PCR) amplification products with primers targeted against the class 1–specific conserved sequences. Lane 1: no template control; lane 2: positive control (In2); lane 3: *Escherichia coli* isolate 16; lane 4: *E . coli* isolate 19; lane 5: *E. coli* isolate 21; lane 6: blank; lane 7: *E. coli* isolate I-6. B: PCR amplification products with primers targeted against the class 2–specific conserved sequences. Lane 1: no template control; lane 2: positive control (In2); lane 3: *E. coli* isolate 29.

**Figure 4:**
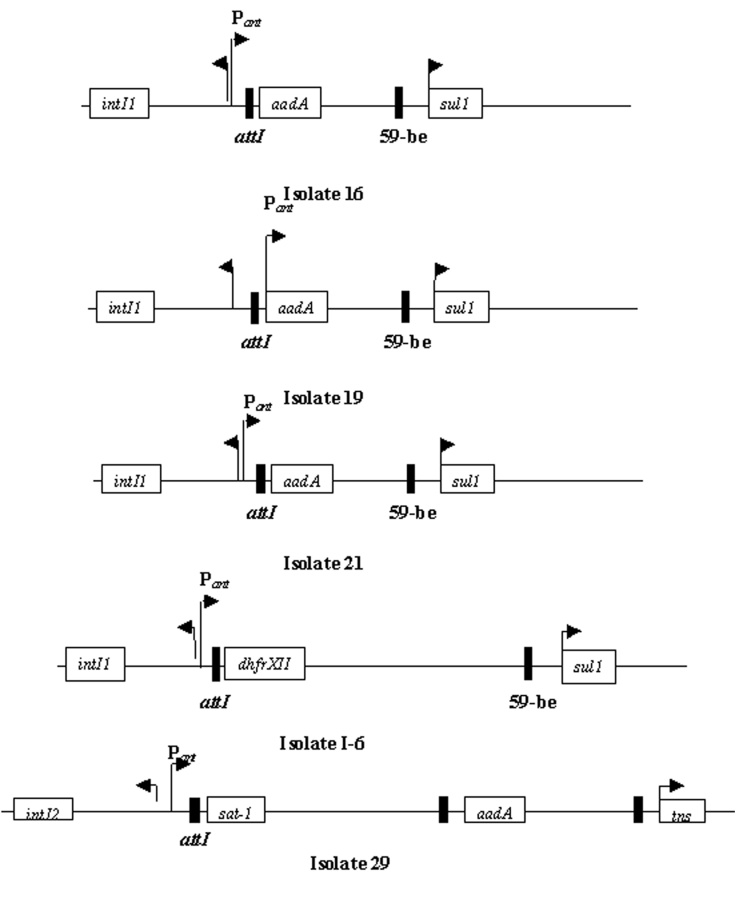
Schematic representations of the four class 1 integrons and one class 2 integron sequenced from multiple antibiotic–resistant *Escherchia coli* isolates.

The 32 isolates identified as multiple antimicrobial resistant were assayed by PCR amplification for class 1 and class 2 integrase genes. Four isolates (approximately 13%) had the class 1 integrase gene *intI1* (Figure 2A), and one isolate had the class 2 integrase gene *intI2* (Figure 2B). Isolates identified as having the class 1 or class 2 integrase genes were further characterized through PCR amplification of the class 1 and class 2 variable regions. Of the four amplified class 1 integron variable regions, three isolates (isolate 16, isolate 19, and isolate 21) were approximately 1 kb in size, but the fourth isolate (isolate I-6) harbored a 2-kb fragment (Figure 3A). The 1-kb amplification products were observed in isolates from the El Paso area. Nucleotide sequencing showed that all of the 1-kb sequences contained a conserved configuration of a 780-bp gene cassette identified as the *aadA* gene (Figure 4). The 2-kb amplification product was seen in an isolate from the Presidio sampling region. Nucleotide sequencing showed that the variable region contained a 498-bp gene cassette, identified as the *dhfrXII* gene, which encodes trimethoprim resistance. The gene cassette did not exhibit perfect homology with the *dhfrXII* gene (Figure 4). Within the identified 498-bp gene cassette, a 323-bp stretch showed 97% sequence homology; in addition, 59-bp and 56-bp fragments showed 88% and 89% homology, respectively. “Islands” of sequence within the variable region showed no sequence homology to any known genes.

When the 32 multiply antimicrobial-resistant isolates were screened for class 2 integrons, only 1 isolate was positive (Figure 2B). This particular isolate (isolate 29) was obtained in the Presidio region and had a 2,600-bp variable region (Figure 3B). Nucleotide sequencing identified two distinct gene cassettes, namely, the *sat-1* and *aadA* genes, which code for streptothricin acetyl transferase, and aminoglycosede adenyltransferase, respectively (Figure 4).

## Discussion

Antimicrobial resistance in human pathogens has become a major public health issue. Resistant organisms have been isolated from a number of natural and man-made environments (*6*,*19*,*20*). In natural environments, resistant organisms can be indigenous or introduced through natural or anthropogenic causes (*21*,*22*). Integron gene sequences have been identified as a primary source of resistance genes and are suspected to serve as reservoirs of antimicrobial resistance genes within microbial populations (*1*,*2*,*23*,*24*). Previous studies along the Texas-Mexico border have shown that fecal contamination of the Rio Grande does occur (*7*,*25*). The isolation of 322 *E. coli* isolates from irrigation water and associated sediments further confirms that fecal wastes are affecting this body of water. Previous studies have reported that municipal and animal wastes regularly harbor multidrug-resistant *E. coli* strains (*6*,*26*,*27*). In this study, 18% of the isolates were resistant to cephalothin. These results are similar to those from a recent survey of U.S. rivers, which found cefotaxime (a third-generation, cephalosporin-resistant, gram-negative bacterium) to range from 16% to 96% across 22 rivers (*19*). The higher frequency of isolation of resistant strains from the El Paso region compared with the other, less urbanized sampling locations is not surprising since the effluent from a number of wastewater treatment plants enters the river at that region (W. McElroy, unpub. data; *28*). Previous studies with sludge and septic tank wastes showed relatively high levels of antimicrobial resistance in *E. coli* (*6*). The precise sources of the *E. coli* isolates used in this study could not be identified because of technical limitations in source tracking (*29*).

Class 1 and class 2 integron gene sequences were found within these *E. coli* isolates. Together, they accounted for 5 (16%) of 32 multidrug-resistant isolates characterized in this study. This prevalence was higher than that reported by Rosser et al. (*30*), who showed that 3.6% of gram-negative bacteria in an estuarine environment contained the class 1 integron. Three of the four class 1 integron-bearing *E. coli* in this study contained the nucleotide sequence of the spectinomycin-streptomycin resistance gene *aadA1* (*31*). Resistance to streptomycin was not observed in these isolates, but resistance to the closely related kanamycin was seen. These results are similar to those reported by Zhao et al. (*3*), who identified that the *aadA* gene transferred to a strain of *Hafnia alvei* but did not report resistance to streptomycin or spectinomycin. These researchers attributed their findings to the inefficient expression of the inserted gene cassette by the integron promoter. Previous studies have also shown that the antimicrobial resistance phenotype can be modulated once these strains are exposed to specific environmental conditions (*32*).

The *aadA* gene cassette is not novel in class 1 integrons. Earlier work by Zhao et al. (*3*) and Bass et al. (*24*) has shown that the *aadA* gene is highly conserved among Shiga toxin–producing and avian clinical *E. coli* isolates, respectively. The only class 2 integron-bearing strain isolated in this study also contained the *aadA* gene in addition to the *sat-1* gene, which codes for resistance to kanamycin, a finding in agreement with the phenotypic expression. The *sat-1* gene, which codes for the streptothricin acetyl transferase, was not detected in any other *E. coli* isolate. The presence of the *sat-1* gene cassette, in combination with the *aadA* gene, suggests that this class 2 integron is likely a derivative of the class 2 integron found on transposon Tn*7* (*33*,*34*).

The *aadA* gene was conserved among the class 1 and class 2 integrons, which suggests a possible selective mechanism for this cassette in enteric bacteria from natural waters. The 2-kb integron-specific variable region–containing strain, which was isolated from the Presidio area, harbored the dihydrofolate reductase gene (*dhfrXII*) instead of the *aadA* gene (*35*).

Overall, these results suggest that the irrigation canals and sediments associated with the Rio Grande are contaminated by bacteria of fecal origin that contain antimicrobial resistance genes. Of 322 *E. coli* isolates, 32 (approximately 10%) were resistant to multiple antimicrobial drugs. Five of these 32 *E. coli* isolates harbored class 1 and class 2 integron sequences. This study did not investigate the possibility that other integron-bearing nonfecal bacteria were present. The occurrence of integron-bearing *E. coli* in irrigation water is important since these organisms are known fecal contaminants, and the potential for lateral gene transfer exists. The results also indicate that integron-bearing strains may not always express the antimicrobial phenotype; thus, phenotype-based isolation of resistant organisms can underestimate the levels of resistant organisms. Studies are needed to identify whether integron-mediated antimicrobial resistance transfer does indeed occur within the irrigation canal sediments and on vegetable surfaces, when they are irrigated with contaminated irrigation water.
